# Identifying Patterns of Primary Care Antibiotic Prescribing for a Spinal Cord Injury (SCI) Cohort Using an Electronic Medical Records (EMR) Database

**DOI:** 10.46292/sci23-00047S

**Published:** 2023-11-17

**Authors:** Arrani Senthinathan, Melanie Penner, Karen Tu, Andrew M. Morris, B. Catharine Craven, Zhiyin Li, Jun Guan, Susan B. Jaglal

**Affiliations:** 1 Institute of Health Policy Management and Evaluation, University of Toronto, Toronto, ON, Canada; 2 KITE (Knowledge Innovation Talent Everywhere), Toronto Rehabilitation Institute - University Health Network, Toronto, ON, Canada; 3 Bloorview Research Institute / Holland Bloorview Kids Rehabilitation Hospital, Toronto, ON, Canada; 4 Department of Paediatrics, Faculty of Medicine, University of Toronto, Toronto, ON, Canada; 5 Department of Family and Community Medicine, University of Toronto, Toronto, ON, Canada; 6 North York General Hospital, Toronto, ON, Canada; 7 Toronto Western Family Health Team, University Health Network, Toronto, ON, Canada; 8 Antimicrobial Stewardship Program, University Health Network, Toronto, ON, Canada; 9 Department of Medicine, Division of Infectious Diseases, Sinai Health, University Health Network, and University of Toronto, Toronto, ON, Canada; 10 Department of Medicine, Faculty of Medicine, University of Toronto, Toronto, ON, Canada; 11 Spinal Cord Rehabilitation Program, Toronto Rehabilitation Institute – University Health Network, Toronto, ON, Canada; 12 ICES, Toronto, ON, Canada; 13 Rehabilitation Science Institute and Department of Physical Therapy, Temerty Faculty of Medicine, University of Toronto, Toronto, ON, Canada

**Keywords:** antibacterial agents, antimicrobial stewardship, primary health care, spinal cord diseases, spinal cord injuries

## Abstract

**Background:**

Individuals with a spinal cord injury (SCI) are considered higher users of antibiotics. However, to date there have been no detailed studies investigating outpatient antibiotic use in this population.

**Objectives:**

(1) To describe primary care antibiotic prescribing patterns in adults with SCI rostered to a primary care physician (PCP), and (2) to identify patient or PCP factors associated with number of antibiotics prescribed and antibiotic prescription duration.

**Methods:**

A retrospective cohort study using linked health administrative and electronic medical records (EMR) databases from January 1, 2013 to December 31, 2015 among 432 adults with SCI in Ontario, Canada. Negative binomial regression analyses were conducted to identify patient or physician factors associated with number of antibiotics prescribed and prescription duration.

**Results:**

During the study period, 61.1% of the SCI cohort received an antibiotic prescription from their PCP. There were 59.8% of prescriptions for urinary tract infections (UTI) and 24.6% of prescriptions for fluoroquinolones. Regression analysis found catheter use was associated with increased number of antibiotics prescribed (relative risk [RR] = 3.1; 95% CI, 2.3-4.1; *p* < .001) and late career PCPs, compared to early-career PCPs, prescribed a significantly longer duration (RR = 1.8; 95% CI, 1.1-3.1; *p* = .02).

**Conclusion:**

UTIs were the number one prescription indication, and fluoroquinolones were the most prescribed antibiotic. Catheter use was associated with number of antibiotics, and PCP's years of practice was associated with duration. The study provided important information about primary care antibiotic prescribing in the SCI population and found that not all individuals received frequent antibiotics prescriptions.

## Introduction

Individuals with a spinal cord injury (SCI) are more likely to experience recurrent infections due to secondary health conditions, pressure ulcers due to mobility issues, and high rates of catheter use.^1^-^10^ These include, but are not limited to, respiratory infections, infected pressure ulcers, and urinary tract infections (UTIs).^1^-^8^ These conditions are usually treated with antibiotics; hence, individuals with SCI may be frequent antibiotic users.^1^-^6^,^10^,^11^ Antibiotic use promotes antimicrobial resistance (AMR).^12^-^14^ Individuals who frequently use antibiotics experience multidrug-resistant bacterial infections, which limits their treatment options and may extend the duration of therapy or number of antibiotics needed to treat an infection.^8^ In addition to AMR, individuals who use antibiotics may experience side effects, such as allergic reactions or diarrhea, which can lead to negative health outcomes such as hospitalizations or emergency room visits.^15^,^16^ Therefore, it is important to optimize antibiotic consumption by ensuring prescribers correctly diagnose bacterial infections and prescribe the right drug, dose, and duration for a given indication.^12^,^17^-^19^

Currently, there is limited published research on antibiotic use in the SCI population. A study conducted among Veterans with SCI in the United States (US) from 2002 to 2007 found an over 5% increase in antibiotic prescription rates in emergency departments over those 5 years, with antibiotics prescribed during 22.8% of visits.^11^ Another study in Veterans with SCI in the US found half of acute respiratory infections resulted in antibiotic prescriptions during ambulatory care visits.^5^ In the United Kingdom (UK), a study found approximately 22% of individuals in an inpatient rehabilitation SCI centre were currently using antibiotics.^15^ A study conducted in Ontario, Canada, investigating prescription drug claims from 2004 to 2014 in older adults hospitalized with traumatic SCI (tSCI), found seven of the top 40 drug classes used by these individuals were antibiotics.^20^ Another study conducted in Ontario investigating prescription drugs dispensed 1 year after inpatient rehabilitation for individuals with nontraumatic SCI (ntSCI) found antibiotics amongst the top four most common drugs dispensed, with 57.8% of individuals receiving antibiotics.^21^ Although these studies suggest antibiotics are frequently prescribed in the SCI population, none of these studies investigated the full range of outpatient antibiotic prescribing for all adults with SCI (i.e., both ntSCI and tSCI).

Although previous literature shows individuals with SCI are regularly prescribed antibiotics, more detailed information on the type of antibiotic classes prescribed, the indications for antibiotic prescriptions, and the duration of antibiotic prescriptions is needed to further understand antibiotic use in the SCI population. In particular, research on previous antibiotic prescribing patterns is needed as there is a substantial time-lag between antibiotic prescribing patterns and resistance patterns. Therefore to address current issues with AMR, research using historical data from the previous decade is needed.^22^,^23^ Prescriber information is also needed as they are responsible for appropriately prescribing antibiotics. There is no published research focusing on primary care physicians (PCPs) regarding antibiotic prescribing in the SCI population. For individuals with SCI, primary care is intended to be the first line for diagnosis and treatment of illnesses, and it has been found that 93% of individuals with an SCI in Canada, the US, and the UK have a PCP.^24^,^25^ Prior to developing any interventions to address antibiotic use for individuals with SCI, investigation of antibiotic prescribing patterns in primary care is needed.

The study purpose is to examine antibiotic prescribing patterns in primary care for adults with SCI in the community and to identify any patient or physician factors associated with primary care antibiotic prescribing. Specific objectives were (1) to describe the number of antibiotics prescribed, antibiotic days of therapy (DOT), type of antibiotic classes prescribed, indications for antibiotic prescriptions, and duration of antibiotic prescriptions; and (2) to identify potential patient or PCP factors associated with number of antibiotics prescribed and duration of individual antibiotic prescriptions.

## Methods

This was a retrospective cohort study conducted using linked health administrative and primary care electronic medical records (EMR) databases at ICES in Ontario, Canada, with data collected between October 2020 and May 2021, for primary care antibiotic prescribing information from January 1, 2013 to December 31, 2015. ICES (formally known as the Institute for Clinical Evaluative Sciences) is an independent, nonprofit research institute whose legal status under Ontario's health information privacy law allows it to collect and analyze health care and demographic data, without consent, for health system evaluation. The use of the data in this project is authorized under section 45 of Ontario's Personal Health Information Protection Act (PHIPA) and was approved by ICES’ Privacy and Legal Office.

### Cohort development

Individuals with SCI were identified in the Electronic Medical Records Primary Care (EMRPC) database at ICES. EMRPC consists of clinical information from PCPs practicing across Ontario derived from their EMR. EMRPC data are derived from approximately 400 PCPs with a total roster of almost 400,000 patients.^26^ EMRPC has data from patient medical charts from April 2010 to March 2016.^26^

A previously tested algorithm (sensitivity = 88.1, PPV = 52.6, F-score = 65.9), developed based on keyword searches conducted in the profile, progress notes, and specialist consult letters sections of the patients’ medical charts, was used to identify potential cases of SCI in EMRPC.^27^,^28^ After potential cases were identified using the algorithm, they were verified through manual chart review before inclusion in the final SCI cohort. To be included in the cohort, cases must have (1) a clear indication of SCI in the clinical notes (an SCI-associated etiology/ cause or a SCI diagnosis by a PCP or specialist) and (2) specific impairments listed in the medical chart. The required impairments for inclusion in the SCI cohort depended on the type of SCI. Impairments based on mechanism of injury has been previously used to identify cases of SCI in health administrative and EMR databases.^28^,^29^ For a tSCI, a sensorimotor, sensory only, bladder or bowel impairment was needed; for a degenerative ntSCI, a sensorimotor impairment was required; and for nondegenerative ntSCI, a sensorimotor or sensory only and a bowel or bladder impairment was required. If the cause of SCI was unclear, then the case was labelled as unclassifiable and had to meet the impairment requirements for all three (tSCI, nondegenerative ntSCI, and degenerative ntSCI). Additionally, to be included in the SCI cohort, the impairments must have been diagnosed before January 1, 2013. To ensure the accuracy of case verification, 10% of cases were re-abstracted. Finally, individuals must have been rostered to a PCP in an EMRPC clinic. Patients rostered to a PCP agreed to try to obtain medical care only from the rostering PCP, and PCPs agreed to provide comprehensive care for their rostered patients.^30^

Clinical and injury characteristics for the finalized SCI cohort were also manually abstracted from EMRPC. This included details about ntSCI versus tSCI, etiology/cause, injury date, catheter use, and impairment(s) (sensorimotor, sensory only, bowel, and bladder). General demographic information for the SCI cohort such as sex, age, and rurality were obtained from the Registered Persons Database (RPDB). The RPDB provides basic demographic information about anyone who has ever received an Ontario Health Insurance Plan (OHIP) card.^26^ Rurality was determined based on individuals’ postal codes and classified based on Rurality Index for Ontario (RIO), with a score for urban less than 10, suburban 10-39, and rural 40 and above.^31^

### Antibiotic prescribing information

A previously developed method, based on key word searches in the medication field of patient charts, was used to identify antibiotics prescribed in EMRPC for this study.^32^ The name of the antibiotic prescribed and the date of antibiotic prescription was outputted from EMRPC into a spreadsheet. Additional antibiotic prescribing information such as indication, duration, and dose was then abstracted by chart reviews. Chart reviews were also done to ensure no duplicate prescription entries on the same day. After abstraction, antibiotics were sorted into the following classes: fluoroquinolones, nitrofurantoin, cephalosporins, penicillins, “sulfonamides trimethoprim & combinations,” macrolides, tetracyclines, clindamycin, metronidazole, and “other.”

The indication for antibiotic prescription was based on the written diagnosis on the EMRPC chart. If the diagnosis was not available on the date of antibiotic prescription, then an indication identified 7 days before or after prescription was used. If multiple indications were found, the diagnosis with the closest date to the antibiotic prescription was used. After the data abstraction, 10% of cases were reviewed to help develop appropriate categories that included (1) UTI (includes bladder-related issues and bacteriuria), (2) pressure ulcers (includes osteomyelitis, cellulitis, deep tissue infections, or abscess), (3) upper respiratory tract infection (URTI; includes sinusitis, pharyngitis, sore throat), (4) lower respiratory tract infection (LRTI; includes chronic obstructive pulmonary disease [COPD], bronchitis, and pneumonia), (4) other (includes tooth infections, eye infections, *Helicobacter pylori* infections, diarrhea, sexually transmitted infections [STIs], and otitis media), and (5) unknown.

For individuals in the SCI cohort, antibiotic prescriptions were calculated as total number of antibiotics prescribed, average number of annual antibiotics prescribed, total antibiotic DOT, and average annual antibiotic DOT during study period. An individual's DOT was calculated by adding together all their antibiotic prescription durations. If a prescription was missing information about duration, the prescription was not included in the DOT calculation.

### Rostered primary care physician description

For the SCI cohort, general demographic information about their rostered PCP from the ICES Physician Database (IPDB) was obtained. The IPDB contains detailed information about physician demographics, main specialty, training, and practice location.^26^ For this study, variables included physician age, sex, rurality of practice, years of practice, and Canadian Medical Graduate (CMG) or non-Canadian Medical Graduate (non-CMG). PCP's years of practice was categorized as early-career (less than 11 years), mid-career (11-25 years), and late-career (over 25 years). These categories were used previously by Fernandez-Lazaro et al. to describe PCPs’ stage of career based on years of practice in another study investigating antibiotic use in the general population.^33^

### Data analysis

Demographic, injury, and clinical characteristics for the SCI cohort and demographic information for the PCP were described using means, standard deviations, and proportions. The difference between groups was calculated using chi-square, *t* test, and Mann–Whitney *U* test. Means, standard deviations, and proportions were also used to describe the primary care antibiotics prescribed for the SCI cohort.

A zero-inflated negative binomial regression was conducted to determine if patient or PCP factors were associated with number of antibiotics prescribed for an individual patient. A negative binomial regression with clustering at patient level was conducted to determine if patient or PCP factors were associated with duration of antibiotic prescription, which accounted for multiple antibiotics prescribed to the same individual, as prescription duration was calculated for each prescription. An assessment of multicollinearity was conducted and factors that exhibited multicollinearity (VIF ≥ 5) were not modeled together in the regression analyses. Only significant factors (*p* < .05) were included in the final regression analysis.^34^,^35^ All data for this study were analyzed with SAS version 9.4 (SAS Institute Inc., Cary, NC, USA; www.sas.com).

## Results

The algorithm identified 931 potential cases of SCI. After manual chart review, 432 cases of SCI (157 tSCI and 275 ntSCI) were included in the SCI cohort for this study. The 10% double abstraction to validate cases had an agreement of 92% and a Cohen's kappa of 0.85 indicating strong agreement.^36^ From January 1, 2013 to December 1, 2015, a total of 1514 antibiotics were prescribed to the SCI cohort in EMRPC. The demographic, clinical, and injury characteristics for the SCI cohort, and details about the number of antibiotics and DOT prescribed, are presented in **Table 1** and **Table 2**, respectively.

**Table 1.** Demographic, injury, and clinical characteristics of SCI cohort clustered by receiving zero versus at least one antibiotic prescription for the study duration (January 1, 2013 to December 31, 2015)

	Individuals with SCI and no antibiotic prescriptions (*n* = 168)	Individuals with SCI and antibiotic prescriptions (*n* = 264)	Full SCI cohort (*n* = 432)
Characteristic^a^	*n* (%)	*n* (%)	*n* (%)
Age at end of 2013			
44 years & under	46 (27.4)	70 (26.5)	116 (26.9)
45-64 years	75 (44.6)	135 (51.1)	210 (48.6)
65+ years	47 (28.0)	59 (22.3)	106 (24.5)
Sex			
Female	60 (35.7)	118 (44.7)	178 (41.2)
Male	108 (64.3)	146 (55.3)	254 (58.8)
Rurality			
Urban	104 (61.9)	164 (60.6)	259 (60.0)
Suburban	34 (20.2)	65 (24.6)	99 (22.9)
Rural	30 (17.9)	35 (13.3)	65 (15.0)
Type of injury			
tSCI & unclassifiable SCI	54 (32.1)	103 (61.0)	157 (35.6)
ntSCI	114 (67.9)	161 (38.3)	275 (63.7)
Duration of injury on			
January 1, 2013*			
Less than 1 year	45 (26.8)	41 (15.5)	86 (19.9)
1-10 years	66 (39.3)	77 (29.2)	143 (33.1)
11-20 years	24 (14.3)	41 (15.5)	65 (15.0)
Over 20 years	33 (19.6)	105 (35.6)	122 (28.2)
Catheter used*			
Yes	49 (29.2)	151 (57.2)	200 (46.3)
No	119 (70.8)	113 (42.8)	232 (53.7)
Impairments			
Sensorimotor	150 (89.3)	225 (85.2)	375 (86.8)
Sensory only	18 (10.7)	39 (14.8)	57 (13.2)
Bladder*	91 (54.2)	206 (78.0)	297 (68.8)
Bowel	47 (28.0)	93 (35.2)	140 (32.4)

*Note:* ntSCI = nontraumatic spinal cord injury; tSCI = traumatic spinal cord injury.

a To avoid small cells (less than six) from being recalculated, missing data for rurality was listed as urban, and missing data for duration of injury was listed as over 20 years. Unclassifiable cases of SCI were listed under the tSCI category.

* Indicates statistically significant (p ≤ .05) difference between individuals who receive no and at least one antibiotic prescription.

**Table 2.** Average annual number of antibiotic prescriptions and antibiotic days of therapy (DOT) for the SCI cohort

	Average annual for SCI cohort (*n* = 432)
**Average annual no. of antibiotic prescriptions, ± *SD***	1.2 ± 2.0
Number of antibiotic prescriptions	***n* (%)**
None	168 (38.9)
1 to 3	214 (49.5)
4 to 6	34 (7.9)
> 6	16 (3.7)
**Average annual DOT for antibiotics,^a^ ± *SD***	22.8 ± 83.3
DOT for antibiotics	***n* (%)**
None	168 (38.9)
1-30	204 (47.2)
31-120	43 (10.0)
>120	17 (3.9)

a Seventeen (1.1%) prescriptions were missing duration, hence were not in DOT counts, underestimating 6 (1.4%) patients’ antibiotic DOT.

The 432 individuals identified in the SCI cohort were rostered to 211 unique PCPs. The average age of PCPs was 44.0 (*SD* 0.9) years, and the average years of practice was 21.8 (*SD* 11.6). Half (50.2%) were female, and 89.6% were CMGs. On average, each PCP had 2.0 (*SD* 1.4) rostered patients with SCI, however 46.9% of PCPs only had one patient with SCI (Q1: 1, median: 2, Q3: 3). It was noted that 84% of antibiotics were prescribed by a patient's rostered PCP, with the rest either prescribed by a nonrostered physician (any PCP that is not the patients rostering PCP) or prescriber information was missing.

Sixty-one percent of individuals in the SCI cohort received at least one antibiotic prescription from their PCP during the study period. For the SCI cohort, 10% of individuals accounted for 48.8% of the total number of antibiotics prescribed. Those who received antibiotic prescriptions had a significantly longer duration of injury and were more likely to use urinary catheters.

**Table 3** outlines the indications and the antibiotic classes prescribed at the patient level and prescription level. The most common indication for antibiotic prescription was UTI (59.8%), and the most common antibiotics class prescribed was fluoroquinolones (24.6%). It was found that 30.1% of individuals received prescriptions for more than one indication, and 37.0% of individuals received prescriptions for more than one antibiotic class during the study period. **Table 4** outlines the average duration of antibiotic prescriptions by antibiotic class and indication.

**Table 3.** Details about prescription indication and antibiotic class at the patient (SCI cohort = 432) and prescription (*n* = 1514) level

	SCI cohort (*n* = 432)^a^	Prescriptions for the SCI cohort (*n* = 1514)
	*n* (%)	*n* (%)
**Indication for prescription**		
UTI	165 (38.2)	905 (59.8)
Pressure ulcer	85 (19.7)	201 (13.3)
URTI	63 (14.6)	100 (6.6)
LTRI	60 (13.9)	106 (7.0)
Other	112 (25.9)	87 (5.7)
Unknown	50 (11.6)	115 (7.6)
**Antibiotic class of prescription**		
Fluoroquinolones	125 (28.9)	372 (24.6)
Nitrofurantoin	101 (23.4)	355 (23.4)
Cephalosporins	99 (22.9)	213 (14.1)
Penicillins	85 (19.7)	200 (13.2)
Sulfonamides, trimethoprim & combinations	84 (19.4)	205 (13.5)
Macrolides	60 (13.9)	101 (6.7)
Tetracyclines	12 (2.8)	26 (1.7)
Clindamycin	13 (3.0)	14 (0.9)
Metronidazole	12 (2.8)	18 (1.2)
Other (HP-Pac^c^, rifampin^b^, fosfomycin^b^)		10 (0.7)

*Note:* LTRI = lower respiratory tract infection; URTI = upper respiratory tract infection; UTI = urinary tract infection.

a Values for SCI cohort do not add up to 100% due to individuals receiving prescriptions for multiple indications and antibiotic classes.

b Since cell count was less than six, information could not be included in the table .

c HP-Pac is used to treat *Helicobacter pylori* and consists of amoxicillin (penicillin) and clarithromycin (macrolide) antibiotics.

**Table 4.** Average duration of antibiotic prescription by indication and select antibiotic class for the SCI cohort

Antibiotic classes	UTI (*n* = 905)		Pressure ulcer (*n* = 211)		LRTI (*n* = 100)		URTI (*n* = 106)	
	*n (*%)	Avg duration ± *SD*	*n (*%)	Avg duration ± *SD*	*n (*%)	Avg duration ± *SD*	*n* %	Avg duration ± *SD*
Fluoroquinolones	274 (30.3)	9.7 ± 10.1	29 (13.7)	10.8 ± 3.0	19 (19)	8.3 ± 2.8	10 (9.4)	9.4 ± 4.5
Nitrofurantoin	327 (36.1)	26.2 ± 56.2	a	a	—	—	—	—
Cephalosporins	57 (6.3)	18.8 ± 53	115 (54.5)	9.1 ± 3.5	8 (8)	7.5 ± 1.7	15 (14.2)	9.0 ± 1.5
Penicillins	82 (9.1)	8.6 ± 2.2	29 (13.7)	32.1 ± 37.5	21 (21)	8.9 ± 1.5	44 (41.5)	9.6 ± 2.7
Sulfonamides, trimethoprim & combinations	161 (17.8)	41.4 ± 91.3	17 (8.1)	12.4 ± 4.6	a	a	a	a
Macrolides	—	—	a	a	46 (46)	13.3 ± 33.0	34 (32.1)	6.6 ± 2.9
Clindamycin	—	—	10 (4.7)	8.7 ± 2.6	—	—	—	—

*Note*: LTRI = lower respiratory tract infection; URTI = upper respiratory tract infection; UTI = urinary tract infection.

a Since cell count was less than six, information could not be included in the table.

**Figure 1.**
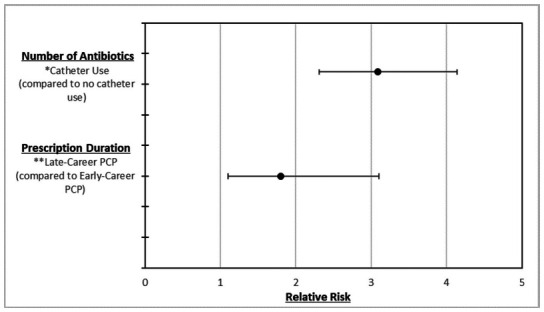
Relative risk for characteristics associated with number of antibiotics prescribed and prescription duration. PCP = primary care physician. *Indicates patient characteristic. ** Indicates physician characteristic.

**Figure 1** outlines the significant factors from the regression analyses for number of antibiotics prescribed and prescription duration. Catheter use was significantly associated with increased number of antibiotics prescribed (RR = 3.1; 95% CI, 2.3-4.1; *p* < .001). Due to multicollinearity between sex, duration of injury, bladder impairment, bowel impairment, sensory impairment, ntSCI versus tSCI, and catheter use, only catheter use was included in the model. Final regression model only included significant factors of catheter use for number of antibiotics. Late-career PCPs, compared to early-career PCPs, were found to prescribe a significantly longer duration (RR = 1.8; 95% CI, 1.1-3.1; *p* = .02). Due to multicollinearity, among physician age and years of practice, only years of practice was included in the model. Final regression model only included significant factors of years of practice for prescription duration.

## Discussion

This study found that not all individuals in the SCI cohort received frequent antibiotic prescriptions, and approximately 40% received no antibiotic prescriptions during the study period. UTIs were the number one indication for antibiotic prescriptions and accounted for nearly 60% of antibiotics prescribed. Nearly a quarter of all antibiotics prescribed were fluoroquinolones, which was the number one antibiotic class prescribed. Those who had a longer duration of injury, used catheters, and had a bladder impairment were more likely to receive at least one antibiotic prescription over the study period. Catheter use was associated with number of antibiotics prescribed, and PCP years of practice was found to be associated with prescription duration.

We could not identify other studies that comprehensively investigated antibiotic classes and indications in adults (above the age of 18) living in the community for both ntSCI and tSCI, making it difficult to compare our study results with previous studies. A previous study in older adults (aged 65 and older) living in Ontario found 40.7% of individuals received at least one antibiotic prescription in a given year from 2006 to 2015.^37^ However, this is less than our finding of 61.1%, which suggests individuals with SCI may receive more antibiotics than older adults. Another study conducted in Ontario using administrative data found 57.8% of individuals with ntSCI received systemic antibiotics 1 year after inpatient rehabilitation in the community,^21^ which is similar to our finding of 61.1%. Another study using administrative data in Ontario from 2004 to 2014 found that approximately 40% of individuals with tSCI over the age of 65 received fluroquinolone antibiotics in the community 1 year after inpatient rehabilitation discharge.^20^ Our study found only 28.9% of individuals received fluroquinolone antibiotics over a 3-year span. The differences in ages and inclusion of ntSCI in our study, as well as differences in prescribing setting, most likely accounts for this variation. Additionally, this previous study was conducted 1 year after discharge from inpatient rehabilitation, whereas our study had over 80% of the SCI cohort over 1 year post-injury.

A key finding in our study was that 38.9% of individuals received no antibiotics prescriptions from their rostered primary care clinics over the 3-year study period. The vast majority of individuals (over 88%) received fewer than four antibiotic prescriptions per year. Furthermore, we found that 10% of individuals accounted for nearly half of antibiotics prescribed. This indicates that only a subset of individuals with SCI are frequent antibiotics users. Therefore, these individuals should be targeted for antibiotic prescribing optimization interventions, as they would be at increased risk of experiencing multidrug-resistant bacteria leading to infective treatment and negative side effects, such as allergic reactions or diarrhea.^3^,^15^,^16^,^38^

Catheter use, the only significant patient factor, in the regression analyses indicated those who use catheters have more antibiotic prescriptions. This is expected as those with bladder issues and catheter use are more likely to have UTIs,^2^,^3^,^7^ which was found to be the number one indication for antibiotic use in this study. It was also found that individuals with longer duration of injury were significantly more likely to receive at least one antibiotic prescription. It is well-established in literature that those with a longer injury duration in the SCI population are at increased risk comorbidities, secondary complications, and negative health outcomes.^39^-^41^ As such, our finding that those with a longer injury duration are more likely to receiving a single antibiotic prescription is in line with previous literature.

Based on the findings from our study, nitrofurantoin and fluoroquinolones were pre-scribed at similar rates at roughly a quarter each of all prescriptions. Previous research found fluoroquinolones were commonly used first line for UTIs and acute exacerbation of COPD in multiple Canadian provinces between 2005 and 2015.^42^ Previous research suggests individuals with SCI exhibit multidrug-resistant organisms, making the selection of appropriate antibiotics difficult.^1^,^3^,^4^,^17^ As such, based on susceptibility test results and other factors, fluoroquinolones may be warranted. Future research should aim to understand appropriateness of fluoroquinolone use in relation to susceptibility testing patterns in the SCI population. Identifying factors that impact the PCP's decision to prescribe certain antibiotic classes over others is also needed.

There are several factors that impact a physician's decision to prescribe antibiotics, however the findings from this study suggest that patient characteristics (catheter use) determine if individuals receive antibiotic prescriptions, whereas physician characteristics may determine the appropriateness of the prescription. The only factor found to be significant for prescription duration in the regression analysis was years of practice. Late-career PCPs, compared to early-career PCPs, prescribed longer antibiotic durations. This is consistent with previous research that found late-career PCPs prescribed longer duration of antibiotics for a general primary care population in Ontario, Canada.^33^ It should be noted that many of the longer antibiotic durations were most likely due to antibiotic prophylaxis, not for treatment of acute infections.

Healthcare providers are the gatekeepers to appropriate antibiotic prescribing. Given that only few individuals with SCI received frequent (greater than three) antibiotics from their PCP, our study indicates that PCP may be judiciously prescribing antibiotics for the SCI population. However, optimal antibiotic prescribing also ensures the right dose, duration, and antibiotic class is prescribed for a given infection.^12^,^17^-^19^,^43^ To avoid AMR, guidelines recommend the shortest clinically safe duration; where possible, narrow-spectrum antibiotics, over broad-spectrum, should be prescribed in individuals with SCI.^44^,^45^ Hence, where possible, PCPs should avoid prescribing broad-spectrum fluoroquinolones antibiotics, which we found was the number one antibiotic class prescribed and accounts for roughly a quarter of all primary care antibiotic prescriptions. Furthermore, PCPs should also avoid longer antibiotic durations, which our study found are more likely to be prescribed by late-career PCPs. Follow-up investigations of the appropriateness of antibiotic prescriptions by PCPs in the SCI population is warranted.

This study is not without limitations. A few cases were missing patient injury characteristics, antibiotic prescribing indications, and duration information. The level of details regarding severity of injury, antibiotic indication, and impairments were also inconsistent across patient charts. For example, it was unclear if a pressure ulcer had progressed to osteomyelitis or other serious complications, which would warrant different antibiotic interventions.^3^ As such, based on the limited information available, the study cannot make comments about appropriateness of the antibiotics prescribed. Similarly based on the information available in charts, we are unable to distinguish between suprapubic catheters and indwelling catheters. Since this article focused on prescribing, it is unclear if individuals consumed the antibiotics they were prescribed. Additionally, despite being rostered to PCPs, individuals may have received antibiotics elsewhere in the community, such as walk-in clinics, emergency rooms, or specialists. It is also uncertain if antibiotics prescribed from the EMRPC databases were from the rostered PCP. However, it was found at least 84% of prescriptions were prescribed by the rostered PCP. The remaining could not be linked to the rostered PCP, because they were either prescribed by a nonrostered physician or prescriber information was missing. Therefore, it can be assumed most antibiotics prescribed were from the rostered PCP. The study was also conducted in individuals rostered to a PCP in Ontario, as such we acknowledge these findings may not be generalizable to individuals with SCI who are not rostered to a PCP. However, previous literature outlines 93% of individuals with SCI had a family physician in Canada, the US, and the UK.^24^ Hence these findings may provide insight into antibiotic prescribing patterns for individuals with SCI in similar health systems as Ontario, Canada.

The information from this study identified key areas for further exploration of primary care antibiotic prescribing for the SCI population. An investigation exploring antibiotic prescribing in the community outside of primary care is also needed. This is the first study to provide detailed information about outpatient SCI population's primary care antibiotic prescribing, such as indications, antibiotic classes, prescription duration, and DOT. This information can inform future research and aid the creation of any potential interventions to optimize antibiotic prescribing in the SCI population. Overall, this study provided important insight about primary care antibiotic prescribing patterns in a rostered primary care SCI population and laid the groundwork for future research.

## References

[1] Suda KJ, Patel UC, Sabzwari R (2016). Bacterial susceptibility patterns in patients with spinal cord injury and disorder (SCI/D): An opportunity for customized stewardship tools. Spinal Cord.

[2] Salameh A, Mohajer MA, Daroucihe RO (2015). Prevention of urinary tract infections in patients with spinal cord injury. Can Med Assoc J.

[3] Abbasi F, Korooni S, Dionyssiotis Y (2018). Essentials of Spinal Cord Injury Medicine.

[4] Heym B, Rimareix F, Lortat-Jacob A, Nicolas-Chanoine MH (2004). Bacteriological investigation of infected pressure ulcers in spinal cord-injured patients and impact on antibiotic therapy. Spinal Cord.

[5] Evans CT (2005). Trends in antibiotic prescribing for acute respiratory infection in veterans with spinal cord injury and disorder. J Antimicrob Chemother.

[6] Evans C, Weaver F, Rogers T (2012). Guideline-recommended management of community-acquired pneumonia in veterans with spinal cord injury. Top Spinal Cord Inj Rehabil.

[7] Skelton-Dudley F, Doan J, Suda K, Holmes SA, Evans C, Trautner B (2019). Spinal cord injury creates unique challenges in diagnosis and management of catheter-associated urinary tract infection. Top Spinal Cord Inj Rehabil.

[8] Evans CT, Fitzpatrick MA, Jones MM (2017). Prevalence and factors associated with multidrug-resistant gram-negative organisms in patients with spinal cord injury. Infect Control Hosp Epidemiol.

[9] Guilcher SJT, Munce SEP, Couris CM (2010). Health care utilization in non-traumatic and traumatic spinal cord injury: A population-based study. Spinal Cord.

[10] Sezer N, Akkus S, Ugurlu FG (2015). Chronic complications of spinal cord injury. World J Orthop.

[11] Evans CT, Rogers TJ, Chin A (2013). Antibiotic prescribing trends in the emergency department for veterans with spinal cord injury and disorder 2002–2007. J Spinal Cord Med.

[12] Dellit TH, Owens RC, McGowan JE (2007). Infectious Diseases Society of America and the Society for Healthcare Epidemiology of America guidelines for developing an institutional program to enhance antimicrobial stewardship. Clin Infect Dis.

[13] O'Neill J (2016). Tackling drug-resistant infections globally: Final report and recommendations.

[14] Nathan C, Cars O (2014). Antibiotic resistance — problems, progress, and prospects. N Engl J Med.

[15] Wong S, Santullo P, O'Driscoll J, Jamous A, Hirani SP, Saif M (2017). Use of antibiotic and prevalence of antibiotic-associated diarrhoea in-patients with spinal cord injuries: A UK national spinal injury centre experience. Spinal Cord.

[16] Bayoumi I, Dolovich L, Hutchison B, Holbrook A (2014). Medication-related emergency department visits and hospitalizations among older adults. Can Fam Physician.

[17] Fitzpatrick MA, Suda KJ, Safdar N (2018). Changes in bacterial epidemiology and antibiotic resistance among veterans with spinal cord injury/disorder over the past 9 years. J Spinal Cord Med.

[18] Barlam TF, Cosgrove SE, Abbo LM (2016). Implementing an antibiotic stewardship program: Guidelines by the Infectious Diseases Society of America and the Society for Healthcare Epidemiology of America. Clin Infect Dis.

[19] Lee CR, Cho I, Jeong B, Lee S (2013). Strategies to minimize antibiotic resistance. Int J Environ Res Public Health.

[20] Guilcher SJT, Hogan ME, Calzavara A (2018). Prescription drug claims following a traumatic spinal cord injury for older adults: A retrospective population-based study in Ontario, Canada. Spinal Cord.

[21] Guilcher SJT, Hogan ME, McCormack D (2021). Prescription medications dispensed following a nontraumatic spinal cord dysfunction: A retrospective population-based study in Ontario, Canada. Spinal Cord.

[22] Nygaard Jensen J, Melander E, Hedin K (2018). Comparison of antibiotic prescribing and antimicrobial resistance in urinary tract infections at the municipal level among women in two Nordic regions. J Antimicrob Chemother.

[23] Bruyndonckx R, Hens N, Aerts M, Goossens H, Cortiñas Abrahantes J, Coenen S (2015). Exploring the association between resistance and outpatient antibiotic use expressed as DDDs or packages. J Antimicrob Chemother.

[24] Donnelly C, McColl MA, Charlifue S (2007). Utilization, access and satisfaction with primary care among people with spinal cord injuries: a comparison of three countries. Spinal Cord.

[25] McColl MA, Aiken A, McColl A, Sakakibara B, Smith K (2012). Primary care of people with spinal cord injury: Scoping review. Can Fam Physician.

[26] ICES Data Dictionary https://datadictionary.ices.on.ca/Applications/DataDictionary/Default.aspx.

[27] Shepherd J (2020). Identifying cases of spinal cord injury/disorder in an ontario primary care electronic medical record database.

[28] Shepherd J, Tu K, Young J (2021). Identifying cases of spinal cord injury or disease in a primary care electronic medical record database. J Spinal Cord Med.

[29] Ho C, Guilcher SJT, McKenzie N (2017). Validation of algorithm to identify persons with non-traumatic spinal cord dysfunction in Canada using administrative health data. Top Spinal Cord Inj Rehabil.

[30] Singh J, Dahrouge S, Green ME (2019). The impact of the adoption of a patient rostering model on primary care access and continuity of care in urban family practices in Ontario, Canada. BMC Fam Pract.

[31] Lucas G, Bielska IA, Fong R, Johnson AP (2018). Rural–urban differences in use of health care resources among patients with ankle sprains in Ontario. Can J Rural Med.

[32] Schwartz KL, Wilton AS, Langford BJ (2019). Comparing prescribing and dispensing databases to study antibiotic use: a validation study of the Electronic Medical Record Administrative data Linked Database (EMRALD). J Antimicrob Chemother.

[33] Fernandez-Lazaro CI, Brown KA, Langford BJ, Daneman N, Garber G, Schwartz KL (2019). Late-career physicians prescribe longer courses of antibiotics. Clin Infect Dis.

[34] Benjamini Y, Hochberg Y (1995). Controlling the false discovery rate: A Practical and powerful approach to multiple testing. J R Stat Soc Ser B Methodol.

[35] Perneger TV (1998). What's wrong with Bonferroni adjustments. BMJ.

[36] McHugh ML (2012). Interrater reliability: The kappa statistic. Biochem Medica.

[37] Tan C, Graves E, Lu H (2017). A decade of outpatient antimicrobial use in older adults in Ontario: A descriptive study. CMAJ Open.

[38] Garcia-Arguello LY, O'Horo JC, Farrell A (2017). Infections in the spinal cord-injured population: A systematic review. Spinal Cord.

[39] Pentland W, McColl MA, Rosenthal C (1995). The effect of aging and duration of disability on long term health outcomes following spinal cord injury. Spinal Cord.

[40] Price GL, Kendall M, Amsters DI, Pershouse KJ (2004). Perceived causes of change in function and quality of life for people with long duration spinal cord injury. Clin Rehabil.

[41] Amsters DI, Pershouse KJ, Price GL, Kendall MB (2005). Long duration spinal cord injury: Perceptions of functional change over time. Disabil Rehabil.

[42] St-Jean A, Chateau D, Dahl M (2021). Regional variation in the potentially inappropriate first-line use of fluoroquinolones in Canada as a key to antibiotic stewardship? A drug utilization review study. BMC Infect Dis.

[43] Crnich CJ, Jump R, Trautner B, Sloane PD, Mody L (2015). Optimizing antibiotic stewardship in nursing homes: A narrative review and recommendations for improvement. Drugs Aging.

[44] Bonkat G, Pickard R, Bartoletti R, Bruyère F, Geerlings SE, Wagenlehner F (2017). EAU Guidelines on Urological Infections.

[45] Kavanagh A, Baverstock R, Campeau L (2019). Canadian Urological Association guideline: Diagnosis, management, and surveillance of neurogenic lower urinary tract dysfunction - Full text. Can Urol Assoc J.

